# Leukemia Inhibitory Factor Enhances Endometrial Stromal Cell Decidualization in Humans and Mice

**DOI:** 10.1371/journal.pone.0025288

**Published:** 2011-09-23

**Authors:** Lorraine Lin Shuya, Ellen Melaleuca Menkhorst, Joanne Yap, Priscilla Li, Natalie Lane, Evdokia Dimitriadis

**Affiliations:** 1 Embryo Implantation Laboratory, Prince Henry's Institute, Clayton, Melbourne, Australia; 2 Department of Anatomy & Developmental Biology, Monash University, Clayton, Melbourne, Australia; Wellcome Trust Centre for Stem Cell Research, United Kingdom

## Abstract

Adequate differentiation or decidualization of endometrial stromal cells (ESC) is critical for successful pregnancy in humans and rodents. Here, we investigated the role of leukemia inhibitory factor (LIF) in human and murine decidualization. *Ex vivo* human (H) ESC decidualization was induced by estrogen (E, 10^−8^ M) plus medroxyprogesterone acetate (MPA, 10^−7^ M). Exogenous LIF (≥50 ng/ml) induced STAT3 phosphorylation in non-decidualized and decidualized HESC and enhanced E+MPA-induced decidualization (measured by PRL secretion, *P*<0.05). LIF mRNA in HESC was down-regulated by decidualization treatment (E+MPA) whereas LIF receptor (R) mRNA was up-regulated, suggesting that the decidualization stimulus ‘primed’ HESC for LIF action, but that factors not present in our in vitro model were required to induce LIF expression. *Ex vivo* first trimester decidual biopsies secreted >100 pg/mg G-CSF, IL6, IL8, and MCP1. Decidualized HESC secreted IL6, IL8, IL15 and MCP1. LIF (50 ng/ml) up-regulated IL6 and IL15 (*P*<0.05) secretion in decidualized HESC compared to 0.5 ng/ml LIF. In murine endometrium, LIF and LIFR immunolocalized to decidualized stromal cells on day 5 of gestation (day 0 = day of plug detection). Western blotting confirmed that LIF and the LIFR were up-regulated in intra-implantation sites compared to inter-implantation sites on Day 5 of gestation. To determine the role of LIF during *in vivo* murine decidualization, intra-peritoneal injections of a long-acting LIF antagonist (PEGLA; 900 or 1200 µg) were given just post-attachment, during the initiation of decidualization on day 4. PEGLA treatment reduced implantation site decidual area (*P*<0.05) and desmin staining immuno-intensity (*P*<0.05) compared to control on day 6 of gestation. This study demonstrated that LIF was an important regulator of decidualization in humans and mice and data provides insight into the processes underlying decidualization, which are important for understanding implantation and placentation.

## Introduction

Successful implantation of a blastocyst and subsequent formation of a functional placenta leading to the establishment of pregnancy in women, is dependent on the adequate decidualization of endometrial stromal cells (ESC) [1]. Decidualization describes the differentiation and proliferation of ESC into morphologically and functionally distinct decidual cells [1]. In women, decidualization is initiated during the mid-late secretory phase by progesterone, independent of the presence of an implanting blastocyst, however the decidua of pregnancy is only formed following blastocyst implantation. In contrast, in rodents decidualization begins only following implantation of a blastocyst into the endometrium of a hormonally primed uterus.

The critical molecular interactions that regulate decidualization are largely unknown, although it has been shown that, in addition to ovarian hormones, locally and temporally produced products such as cytokines and growth factors (eg. interleukin [IL] 11, relaxin, prostaglandin E2, activin A, and corticotrophin-releasing hormone) progress decidualization [2].

Leukemia inhibitory factor (LIF), an IL6-type cytokine, is one of a very few cytokines shown to be critical for implantation in mice [3]. LIF mRNA and protein is maximally expressed in the murine endometrial glandular epithelium just prior to blastocyst implantation [4], [5]: LIF^−/−^ female mice are infertile due to implantation failure: blastocysts cannot adhere to the endometrial luminal epithelium [6].

Likewise in women, LIF is maximally expressed by the luminal and glandular epithelium during the mid-secretory phase of the menstrual cycle, the period during which the uterus is receptive to an implanting blastocyst [3]. Lower levels of LIF are found in uterine flushings from some infertile women compared to fertile women [3] and we have demonstrated that LIF regulates the adhesive properties of human endometrial epithelial cells [7] supporting a role for LIF in human blastocyst attachment to the endometrium, similar to mice.

Whether LIF has a role in decidualization is not clear. In women, LIF protein is present in the endometrial stroma during the mid-late secretory phase of the menstrual cycle, but at considerably lower levels than the epithelium [3]. During pregnancy, LIF mRNA and protein are expressed in the first trimester decidua [3]. In the only *in vitro* study using human (H) ESC, exogenous LIF had no effect on 8-bromo cyclic adenosine monophosphate (cAMP) analog induced decidualization [8], however it is not known whether LIF has a role in progesterone induced decidualization. Certainly, both the progesterone and cAMP pathways are required for decidualization [9], however progesterone rather than cAMP is the main physiological inducer of decidualization in vivo; although cAMP may ‘prime’ HESCs to the action of progesterone [10]. Further, cAMP and progesterone may use different pathways during decidualization [10], [11]. Additionally, other cytokines have been shown to progress progesterone induced decidualization whilst having no effect on cAMP induced decidualization [12], [13].

The role of LIF in murine decidualization is also unclear. Unlike in women, in mice decidualization of ESC occurs post-implantation. LIF^−/−^ female mice do not undergo artificial decidualization [14] and intraluminal administration of a short-acting LIF inhibitor during the peri-implantation period results in less extensive desmin filaments (decidual marker) than in the control mice [15]. Further, intraluminal injections of LIF into Fox2a null females partially rescues the formation of a deciduoma during artificial decidualization [16]. Conversely however, LIF inhibits decidualization of murine stromal cells *in vitro* [17]. We have previously used a long-acting, polyethylene glycol (PEG) conjugated LIF antagonist (PEGLA) to block LIF action in the endometrial luminal epithelium during the peri-implantation period in mice. This resulted in implantation failure [18], [19] replicating the LIF^−/−^ phenotype [6], however the effect of LIF inhibition during the initiation of decidualization is unknown.

Here we aimed to investigate whether LIF has a role in decidualization in humans and in mice and to define the mechanism by which this may occur. Specifically, we aimed to determine whether LIF could alter progesterone induced decidualization of HESC *ex vivo*, and to determine the effect of LIF inhibition on *in vivo* decidualization in mice using a long-acting LIF antagonist (PEGLA).

## Materials and Methods

### Ethics Statement

#### Human ethics

Written informed consent was obtained from each patient and the study was approved by the Southern Health Research and Ethics Committee (#09317B; #06014C) at Monash Medical Centre Melbourne, Australia.

#### Animal ethics

All procedures were approved by the Monash Medical Centre Animal Ethics Committee (#MMCB2007/21) and followed the NHMRC Australian Code of Practice for the Care and Use of Animals for Scientific Purposes.

### Human tissue collection

Endometrial biopsies were collected from women with regular menstrual cycles between days 8–24. The women had no steroid treatment for at least 2 months prior to tissue collection. The biopsies were examined by an experienced gynaecological pathologist to confirm that they had no apparent endometrial dysfunction. Normal 1^st^ trimester decidual tissue was collected from healthy women undergoing elective termination of pregnancy (amenorrhea: 7–11 weeks). Endometrial and decidual biopsies were either fixed in 10% neutral buffered formalin for 18 h and processed to wax or placed in Dulbecco's Modified Eagle's Medium/F12 (DMEM/F12 GIBCO® Invitrogen, Mt Waverly, Vic, Australia).

### LIF and LIFRα immunohistochemistry in human endometrium

Paraffin-embedded, formalin-fixed endometrial tissue from the mid-late secretory phase of the menstrual cycle and 1^st^ trimester decidua (n = 4–6 per group) were dewaxed in histosol and rehydrated in ethanol. LIF was immunolocalized as previously described [20] except that the primary antibody was incubated overnight at 4°C and a goat anti-rabbit secondary (Vector, Vector Laboratories Inc, Burlingham, California, USA) was used. LIF receptor αLIFR) was immunolocalized as follows: endogenous hydrogen peroxidase activity was quenched using 3% H_2_O_2_ in methanol for 10 mins at room temperature. Sections were blocked in non-immune serum (10% horse, 6% fetal calf and 2% human serum in 0.1%Tween-20 Tris-buffered saline [TBS]) for 1 hr at room temperature (RT) before the primary antibody (LIFRα, 2.5 µg/ml, #AF-249-NA R&D Systems) was applied for 1 h and incubated at RT. A non-immune goat IgG isotype control diluted to a matching concentration as the primary antibody was included. After stringent washing with 0.6% Tween 20 in TBS, biotinylated horse anti-goat secondary antibody (1∶200, Vector) was applied for 30 min at RT followed by a 30 min incubation with streptavidin-biotin complex/HRP (Vector) before sections were stained with the substrate 3′3-diaminobenzidine (K3466, DAKO). Quality controls were included in each run.

### HESC in vitro decidualization

HESC were isolated from tissue by enzymatic digestion and filtration as previously described [13], [21], [22]. HESC isolated by this method are 97% pure as assessed by immunostaining for cytokeratin and vimentin [13]. Cells were plated in 25 cm^2^ flasks or 12 well plates (NUNC, In Vitro technologies, Noble Park North, VIC, Australia) and grown to confluence. Once confluent, HESC were cultured overnight in low serum media (DMEM/F12+2% charcoal stripped fetal calf serum [FCS], 1% antibiotics and antimycotic) to suppress the production of any endogenous factors. Decidualization was conducted in low serum media to minimize cell proliferation. Cells were treated with 10^−8^ M estradiol 17β (E; Sigma Chemical Co., St Louis, MO, USA) plus 10^−7^ M medroxy-progesterone acetate (MPA; Sigma) for 14 days. The media containing treatments was replenished every 48 h and supernatant was collected, centrifuged at 160×*g* to pellet any non-adherent cells and stored at −20°C. On Day (D) 14, cells were washed twice with ice-cold sterile Phosphate Buffered Saline (PBS, calcium and magnesium free) before being lysed in 200 µl ice-cold Universal immunoprecipitation (UIP) lysis buffer (50 mM Tris base, 150 mM NaCl, 2 mM EDTA, 2 mM EGTA, 25 mM NaF, 0.2% TritionX-100, 0.3% Nonidet P-40, 25 mM ß-glycerolphosphate [pH 7.5]) containing Protease Inhibitor Mixture Set III (1∶500; Calbiochem, San Diego, CA), centrifuged at 9660×*g* to pellet cell membrane and cell debris. Supernatants containing the protein lysate were assayed for total protein using the BCA Protein Assay Kit (Pierce, Quantum Scientific, Rockford, IL).

Prolactin (PRL) secretion is used to measure the degree of *in vitro* HESC decidualization [23]. Supernatant from decidualizing HESC was concentrated 10-fold overnight (SpeediVac SC100, Savant, GMI Inc., Minneapolis, USA) and PRL secretion measured quantitatively by ELISA (Bioclone Australia Pty Ltd., Marrickville, NSW, Australia) according to the manufacturer's instructions. The lower detection limit of this assay is 50 mIU/L and the inter- and intra-assay variabilities were 5.3 and 3.0%, respectively. PRL secretion was normalized to total cellular protein.

### LIF and LIFR mRNA expression in non-decidualized and decidualized HESC

Total RNA was isolated from cultured HESC (media alone, E, E+MPA-treated; n = 4/group) using the RNeasy Minikit (QIAGEN Sciences, Germantown, Maryland, USA) according to the manufacturer's instructions. Genomic DNA was digested using the DNAfree kit (Ambion) according to the manufacturer's instructions. RNA samples were analysed by spectrophotometry (Nanodrop, Perkin Elmer, Waltham, Massachusetts, USA) at an absorbance ratio of A260/280 nm to determine RNA concentration, yield and purity. cDNA was synthesized from total RNA (500 ng) using Superscript III reverse transcriptase (Invitrogen) and analyzed by spectrophotometry at an absorbance ratio of A260/280 nm to determine concentration and purity.

#### Polymerase chain reaction

PCR reactions were performed using PCR express machine (Thermo Fisher Scientific Inc., Milfred, MA, USA) and GoTaq master mix (Promega) according to the manufacturer's instructions. HESC cDNA was analysed for LIF, LIFR and 18 s using reaction conditions of an initial denaturation at 95°C for 5 mins, followed by 30 (LIF, LIFR) or 22 (18 s) cycles of: denaturation, 94°C for 1 min; annealing, 55°C for 1 min (LIF, 18 s) or 65°C for 30 s (LIFR); extension, 72°C for 1 min; with a final extension at 72°C for 10 min. Primers used were LIF (115 base pairs) [24] fwd 5′-TGA ACC AGA TCA GGA GCC T-3′, rev 5′-CCA CAT AGC TTG TCC AGG TTG TT-3′; LIFR (356 base pairs) [24] fwd 5′-GTG GCA GTG GCT GTC ATT GTT GGA GTG GT-3′, rev 5′-TCA TCT GCG GCT GGG TTT GGT ATT TCT TC-3′; 18 s (187 base pairs) fwd 5′- GAT CCA TTG GAG GGC AA GTC T-3′, rev 5′-CCA AGA TCC AAC TAC GAG CTT TTT-3′. The PCR products were run on a 1% agarose gel with 1000 bp DNA ladder (Invitrogen) to semi-quantify LIF and LIFR expression.

### LIF activation of STAT3 in HESC

#### Treatments

Confluent decidualized (D) or non-decidualized (ND) HESC (25 cm^2^ flask; cultured from individual endometrial biopsies, n = 3 women/group; ND incubated overnight in low-serum media) were treated with LIF (5, 50, 100 and 200 ng/ml; R&D systems) in serum-free media for 15 min. HESC were also treated with a LIF antagonist (LA; a kind gift from Drs Nick Nicola and Jianguo Zhang, Walter and Eliza Hall Institute) [25] for 30 min prior to the addition of LIF (100 ng/ml).

#### pSTAT3/STAT3 Western blot

After LIF treatment the medium was aspirated and cells were washed twice with ice cold sterile PBS before being lysed as described above. Total cellular protein (25 µg) was resolved on an 4–11% SDS/PAGE gel, then transferred to Hybond-P PVDF membranes (GE healthcare, Amersham, UK) before the membranes were probed for pSTAT3 and STAT3 (both Cell Signaling Technology) and densitometry performed as previously described [26].

### Effect of exogenous LIF on decidualization

HESC were induced to decidualize as described above. The effect of LIF on decidualization (PRL secretion) was examined by including exogenous human recombinant LIF (5, 50, 100 and 200 ng/ml; R&D systems) to the decidualization treatment (n = 5/group). Further, the effect of LIF inhibition on decidualization was investigated by the addition of LA (10 µg/ml) from D8 of treatment (50 ng/ml) in decidualizing HESC (n = 3).

### Cytokine secretion by 1^st^ trimester decidua parietalis and decidualizing HESC

The secretion of specific cytokines by decidua parietalis biopsies (decidua containing no extravillous trophoblast) or by decidualizing HESC with LIF treatment (0.5 and 50 ng/ml) was investigated using the Multiplex 12-plex Human Cytokine Assay Kit (Millipore, MA, USA) following the manufacturer's instructions. The kit included FGF2, G-CSF, TNF-α, GM-CSF, IL-1β, IL-1rα, IL-6, IL-8, IL-10, IL-15, MCP-1 and VEGF. The detection range of the cytokines was from 3.2 pg/ml to 10,000 pg/ml. Data analysis of samples and standards was performed by Bio-Plex manager software version 4.0.1. For statistical analysis the multiplex data was normalized to protein concentration and also prolactin secretion to account for the differences in the degree of decidualization found between cultures isolated from different women and because LIF itself promoted decidualization thus enhanced decidualization itself may have promoted cytokine secretion, not LIF.

#### Decidual biopsy conditioned media

Decidual tissue (n = 3) was cut into thin squares (∼2 mm^3^), and placed on top of siliconised lens tissue paper [27] floating on 1 ml of serum-free DMEM/F12 and cultured for 72 h at 37°C with 5% carbon dioxide. Explant culture on siliconised lens paper facilitates both the absorption of required nutrients from the medium and gas exchange [27]. After culture, the conditioned media was collected and concentrated 10-fold as described above. Representative decidual explants were examined by histology for evidence of necrosis and human leukocyte antigen (HLA) G^+^ extravillous trophoblast (data not shown).

#### HESC cell conditioned media

The conditioned media for HESC was collected from HESC induced to decidualize as described above in the presence of LIF (0.5 or 50 ng/ml) for 48 h (n = 5).

### Inhibition of LIF action during early decidualization in mice

#### Animals

Female (virgin 8–12 weeks old) and male C57BL/6J mice (Monash Animal Services, Clayton, Australia) were housed under conventional conditions, with food and water available *ad libitum* and held in a 12 hr light and dark cycle.

#### Immunohistochemistry for LIF and LIFR

Inter- and intra-implantation sites were collected from mated female mice treated with 1000 µg PEG on Day (D) 5 of gestation (D0 = day of plug detection) and either fixed (<24 h) for histology in 10% neutral buffered formalin or snap frozen.

Paraffin embedded, formalin fixed mouse implantation sites (n = 3 per group) were dewaxed and rehydrated in ethanol. LIF and LIFR were immunolocalised as described above except that the non-immune block used was: LIF: 10% normal goat plus 2% normal mouse serum and LIFR: 10% normal horse and 2% mouse serum; and two LIF antibodies were used to confirm the specificity of LIF staining (rabbit anti-human LIF antibody as above and in [20] and goat anti-human LIF antibody, R&D systems; 10 µg/ml).

#### LIF ELISA

Frozen intra- and inter-implantation sites (n = 3/group) were homogenized and cells lysed in UIP lysis buffer as described above. Total cellular protein (20 µg) was added to each well of the Human LIF ELISA (ELH-LIF-001; RayBiotech) and LIF detected according to the manufacturer's instructions.

#### Western blotting for LIFRα

Frozen intra- and inter-implantation sites (n = 3/group) were homogenized and cells lysed in UIP lysis buffer as described above. Total cellular protein (20 µg) was resolved on an 4–11% SDS/PAGE gel, then transferred to Hybond-P PVDF membranes (GE healthcare, Amersham, UK) before the membranes were blocked in 3% bovine serum albumin (BSA) and probed for LIFR (1∶1000; R&D Systems) and β-actin (1∶2000, Cell Signalling Technology, HRP-conjugated) overnight at 4°C or 1 h at RT. After washes in TBS and TBS 0.1% Tween, the LIFR blot was incubated with the secondary antibody (rabbit anti-goat, 1∶4000, DAKO; β-actin, sheep anti-mouse, Amersham) for 1 h at RT before antibody binding was detected using Pierce ECL Western Blotting Substrate (Thermo Scientific).

#### PEGLA treatment to block decidual LIF action

Polyethylene glycol (PEG) conjugated LIF-antagonist (PEGLA) [19] and PEGylation reagent control were a kind gift from Drs Nick Nicola and Jianguo Zhang (Walter and Eliza Hall Institute, Parkville, Victoria, Australia). The LIF antagonist (LA) binds to the LIF receptor but does not bind to the LIF receptor complex signalling component, gp130, preventing the initiation of downstream signalling. LA was covalently bound to PEG (PEGLA) to increase the period of sera retention [19]. PEGLA acts on the uterus to block implantation when delivered by intra-peritoneal (IP) injection during the peri-implantation period [19].

To inhibit LIF action in the endometrial stroma during early decidualization, mated female mice received a single IP injection of 900 or 1200 µg (150 or 200 mM; optimised from initial studies; n = 2–3/group) PEGLA or PEG control (equivalent molarity) at 10am on D4 of gestation immediately post-implantation which begins at D3.5 [28] and mice were killed on D6 of pregnancy when the implantation sites are clearly visible. Uterine horns were removed and the number of implantation sites and corpora lutea counted. Implantation sites were dissected out and fixed in 10% neutral buffered formalin (<18 hrs).

#### Desmin immunohistochemistry

Paraffin-embedded, formalin-fixed implantation sites were dewaxed in histosol and rehydrated through ethanol to water. Endogenous hydrogen peroxidase activity was quenched using 3% H_2_O_2_ in methanol for 10 min in the dark at RT before being washed with high salt TBS (300 mM NaCl, 5 mM TrisCl in distilled water pH 7.6). Sections were blocked in non-immune serum (15% normal goat serum 0.1%Tween-20 high salt TBS) for 30 min at RT before primary antibody was applied (Desmin, 2.9 µg/ml, M0760, DAKO; negative: mouse IgG 2.9 µg/ml DAKO) in block (10% normal goat serum in 0.1% Tween-20 high salt TBS) and incubated for 30 min at RT. After stringent washing (2×5 min) with 0.6% Tween-20 in high salt TBS, the Envision^+^ system labelled Polymer-HRP anti-mouse (K4001, DAKO) was applied to sections for 30 m at RT. Sections were washed twice with 0.6% Tween-20 in high salt TBS and then twice with high salt TBS before the substrate 3′3-diaminobenzidine (K3466, DAKO) was applied. Quality controls were included in each run. Immunostaining was analyzed semiquantitively by two independent and blinded observers as previously described [26]. The intensity of desmin staining in the decidua was assessed and allocated a score between 0 (no staining) and 3 (strong staining) relative to positive and negative controls. The area of desmin staining was quantified using Motic Images Plus 2.0 software (Motic China Group Co Ltd).

### Statistical analysis

GraphPad Prism 5.0 Windows (GraphPad Software, San Diego, CA, USA) was used for all statistical analyses. Statistical advice was obtained from Dr. Aidan Sudbury (statistician) at Monash University. PRL secretion data was normalized to total cellular protein and analyzed by the non-parametric Kruskal Wallis test. Non-parametric t-test (Mann Whitney) was used to compare the secretion of cytokines by decidualizing HESC. The weight, size of decidual area and desmin intensity staining in mouse implantation sites were compared by student's t-test. All results are given as mean ± standard error of the mean (SEM). A P value of <0.05 was considered significant.

## Results

### LIF and LIFR immunolocalized to decidual cells in the mid-late secretory phase endometrium and 1^st^ trimester decidua

**Figure 1:**
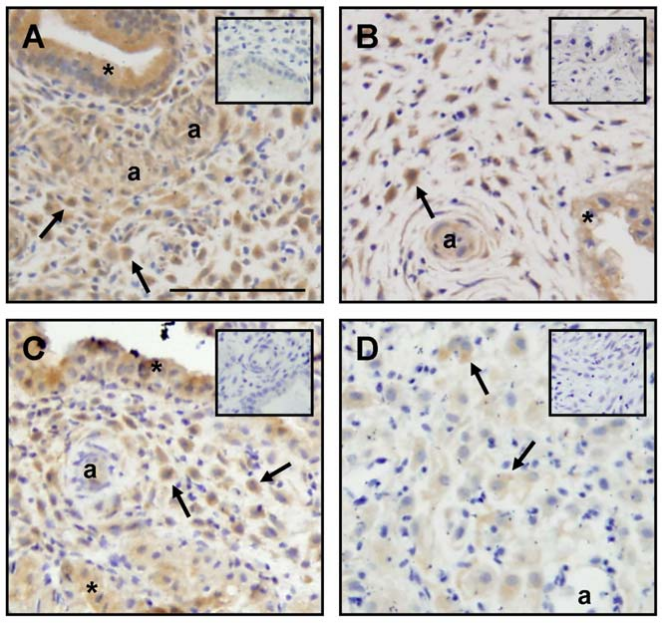
LIF and LIFR immunolocalization in human endometrial biopsies. A. LIF immunolocalized to glandular epithelium (*), endothelial cells (a) and decidualized stromal cells (arrows) during the mid-secretory phase. B. LIF immunolocalized to decidual cells (arrows), endothelial cells (a) and glandular epithelium (*) in 1^st^ trimester decidua. C. LIFR immunolocalized to glandular epithelium (*) and decidualized stromal cells (arrows) during the mid-secretory phase. D. LIFR immunolocalized to decidual cells (arrows) in 1^st^ trimester decidua. Representative photomicrographs from n = 3. Scale, 100 µm; Insert, negative (IgG) control; a, spiral arterioles.

Endometrial epithelial cells were a major source of LIF immunoreactivity (Figure 1A). LIF immunoreactivity was also found in the endometrial stroma during the mid-late secretory phase, particularly in decidualized stromal cells located near spiral arterioles and endothelial cells (Figure 1A). In first trimester decidua, LIF immunoreactivity was strong in decidual cells and present in endothelial cells and glandular epithelium (Figure 1B).

The LIFR localised to the glandular epithelium and in decidualized stromal cells in the mid-late secretory phase endometrium and in first trimester decidua (Figure 1C & D).

### LIF and LIFR expression following decidualization stimulus

**Figure 2:**
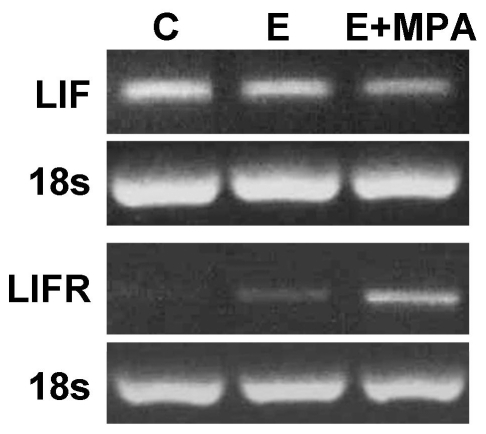
LIF and LIFR mRNA expression in non-decidualized (media control [C] and estradiol [E] treated) and decidualized (E plus medroxy-progesterone acetate [MPA] treated) HESC determined by semi-quantative reverse-transcription PCR. 18 s ribosomal RNA was used as loading control. LIF expression was downregulated by E+MPA, whilst LIFR expression was stimulated by both E and particularly E+MPA. Representative gel of n = 5.

LIF mRNA expression in HESC was down-regulated following treatment with E+MPA compared to E or media control at D14 of decidualization treatment (Figure 2). LIFR mRNA was expressed following treatment with E alone and was up-regulated following treatment with E+MPA (Figure 2) compared to control and E groups at D14 of decidualization treatment.

### LIF activated STAT3 in non-decidualized and decidualized HESC

**Figure 3:**
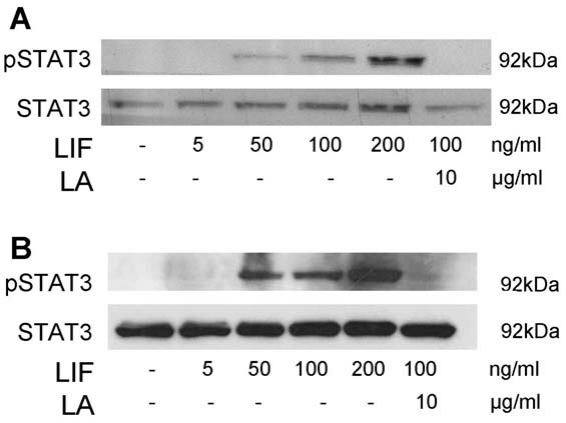
Leukemia inhibitory factor (LIF) activated STAT3 in HESC. Cells were treated with LIF for 15 min and with a LIF antagonist (LA) for 30 min prior to the addition of LIF. **A.** Representative immunoblot for pSTAT3 and total STAT3 in cell lysates from non-decidualized HESC (n = 3). **B.** Representative immunoblot for pSTAT3 and total STAT3 in cell lysates from decidualized HESC (n = 3).

The effect of LIF on STAT3 phosphorylation (pSTAT3) in non-decidualized (ND) and decidualized (D) HESC was examined by Western blot (Figure 3A & B). pSTAT3 was not detectable in ND or D HESC cultured under 2% serum conditions (Figure 3A & B). Addition of LIF (≥50 ng/ml) stimulated pSTAT3 abundance in both ND and D HESC in a concentration-dependent manner (Figure 3A & B) compared to respective control. Co-incubation of LIF (100 ng/ml) plus LA (10 µg/ml) diminished STAT3 activation in ND and D HESC compared to LIF (100 ng/ml) alone respectively (Figure 3A & B). STAT3 protein abundance was not affected by LIF or/and LA treatment (Figure 3A & B) in both ND and D HESC.

### LIF enhanced HESC decidualization

**Figure 4:**
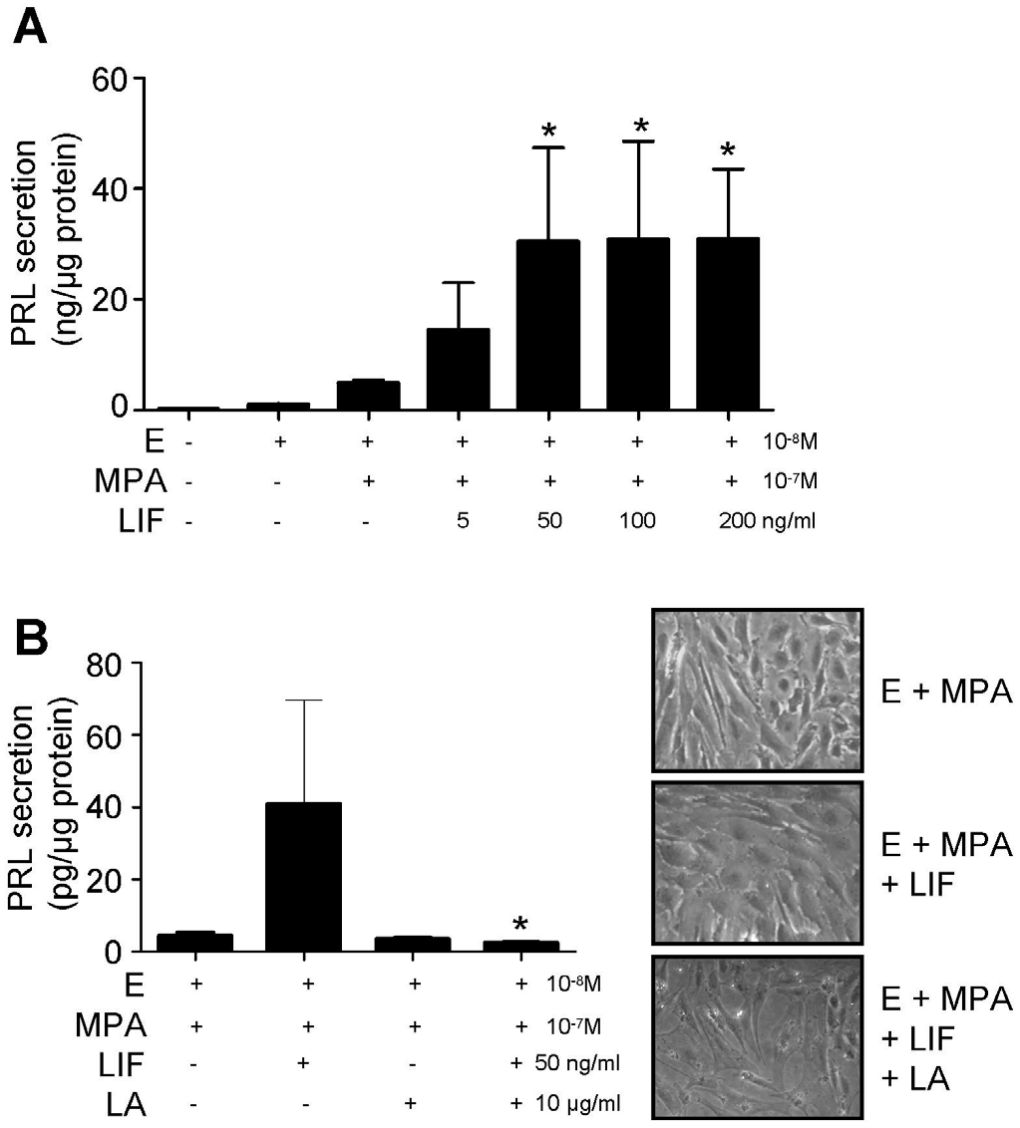
Exogenous LIF enhanced estradiol (E) + medroxy-progesterone acetate (MPA)-induced *in vitro* decidualization (measured by PRL secretion) in HESC. **A.** LIF treatment significantly (*; p<0.05) promoted E+MPA-induced decidualization in HESC compared to E+MPA-treated control on D14 (n = 5). **B.** Co-incubation of E+MPA plus LIF and LIF antagonist (LA) significantly (*; p<0.05) inhibited HESC decidualization compared to HESC treated with E+MPA plus LIF (n = 3). Treatments: E 10^−8^ M; MPA 10^−7^ M.

HESC treated with media or E alone did not secrete detectable levels of PRL on D14 (Figure 4A). HESC treated with E+MPA secreted PRL on D14 (4.9±0.6 ng PRL/µg protein; Figure 4A). PRL secretion by E+MPA-treated HESC (decidualizing) was significantly enhanced following treatment with ≥50 ng/ml exogenous LIF (50 ng/ml, 30.4±17; 100 ng/ml 30.8±17.7; 200 ng/ml, 30.9±12.6 ng PRL/µg protein; Figure 4A; Kruskal-Wallis statistic 27.73, df 7, p<0.05).

The specificity of LIF action was confirmed by co-treatment of decidualizing HESC with LIF (50 ng/ml) +/− LA (10 µg/ml; Figure 4B). Co-incubation of decidualizing HESC with LIF plus LA reduced PRL secretion to levels found in E+MPA alone on D14 (E+MPA, 3.9±1.2; E+MPA+LIF, 41.0±28.6; E+MPA+LIF+LA, 2.6±0.1 pg PRL/µg protein; Figure 4B; Kruskal-Wallis statistic 9.462, df 4, p<0.05). Treatment of decidualizing HESC with LA alone (no LIF treatment) to block endogenous LIF had no effect on decidualization (E+MPA, 4.4±0.8; E+MPA+LA, 3.6±0.3 pg PRL/µg protein; Figure 4B).

### LIF (50 ng/ml) modified cytokine secretion by decidualizing HESC

**Figure 5:**
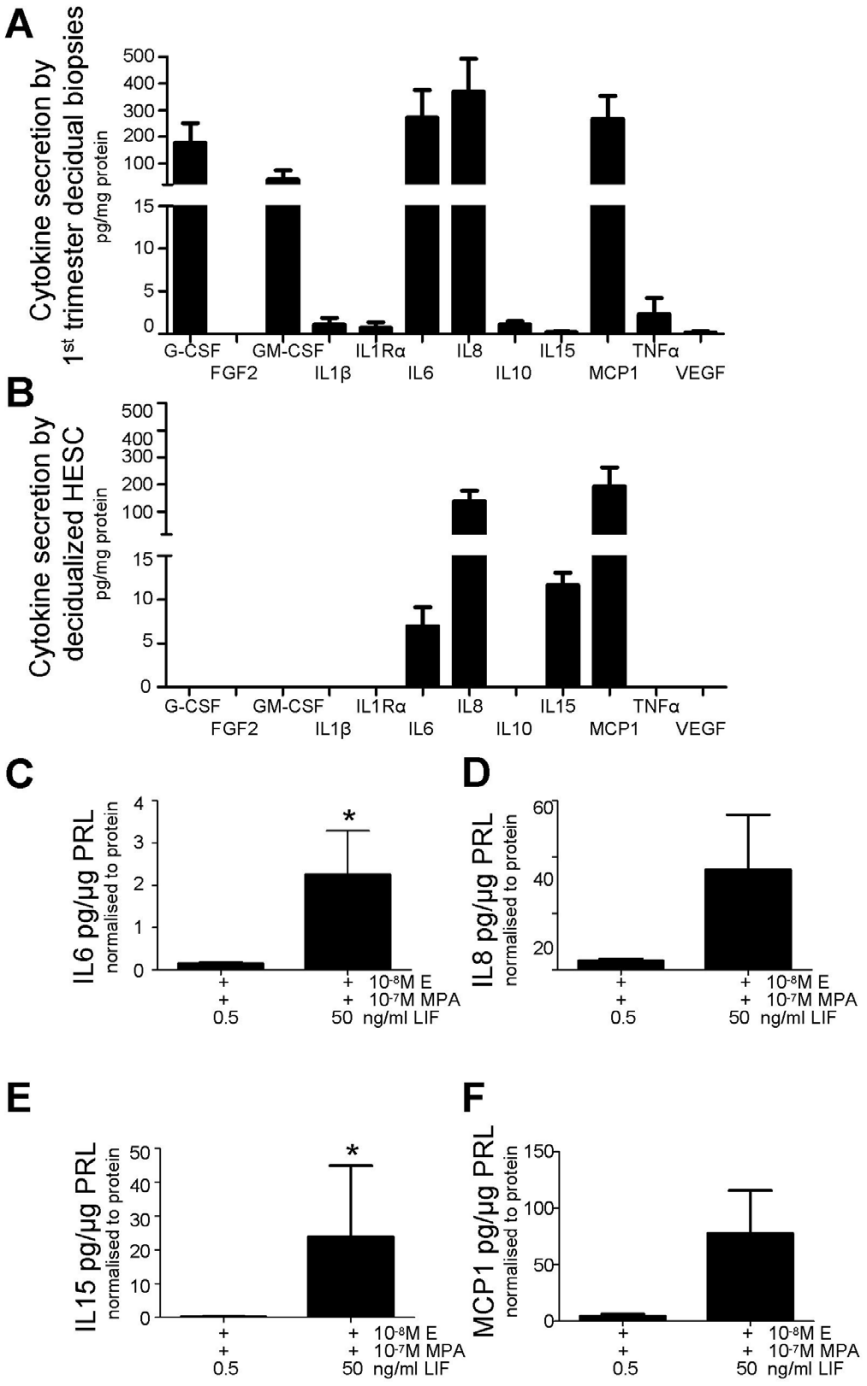
LIF (50 ng/ml) enhanced IL6 and IL15 secretion by *in vitro* decidualized HSEC. A. Cytokine secretion by first trimester decidual biopsies (n = 3). B. Cytokine secretion by HESC decidualized with estradiol (E) and medroxy-progesterone acetate (MPA) (n = 5). C. LIF treatment (50 ng/ml) significantly (*; compared to 0.5 ng/ml; p<0.05) induced IL6 secretion by decidualized HESC (n = 5). D. LIF treatment (50 ng/ml) had no significant effect (compared to 0.5 ng/ml; p = 0.222) on IL8 secretion by decidualized HESC (n = 5). E. LIF treatment (50 ng/ml) significantly (*; compared to 0.5 ng/ml; p<0.05) induced IL15 secretion by decidualized HESC (n = 5). F. LIF treatment (50 ng/ml) had no significant effect (compared to 0.5 ng/ml; p = 0.222) on MCP1 secretion by decidualized HESC (n = 5).

First trimester decidual biopsies secreted detectable levels of all cytokines examined except FGF2 (Figure 5A). The decidual biopsies secreted high levels of G-CSF, GM-CSF, IL6, IL8 and MCP1 (Figure 5A). Decidualized HESC secreted detectable levels of only IL6, IL8, IL15 and MCP1 (Figure 5B). Addition of 50 ng/ml LIF to the decidualizing HESC increased the secretion of IL6 and IL15 compared to 0.5 ng/ml LIF (IL6, 0.5 0.2±0.1, 50 2.3±1.0 pg/µg PRL/µg protein; Mann-Whitney U 2.0, p<0.05; IL15, 0.5 0.3±0.1, 50 23.9±20.9 pg/µg PRL/µg protein; Mann-Whitney U 0.0, p<0.05). The trend was similar for IL8 and MCP1 but did not reach statistical significance (Figure 5C–F).

### LIF and LIFR immunolocalized to mouse implantation site

**Figure 6:**
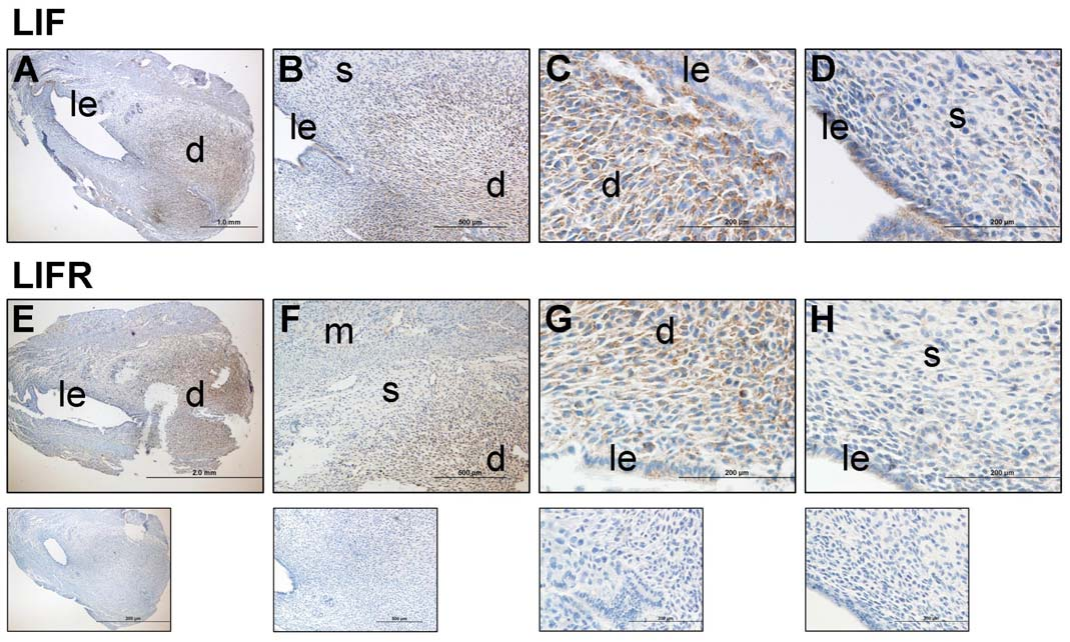
LIF and LIFR immunolocalized to decidualizing stromal cells on Day 5 of gestation in mice. **A–D.** LIF immunolocalized strongly to the decidua (d) and also to the luminal epithelium (le). **E–H.** LIFR immunolocalized strongly to the decidua. Bottom row, negative controls to sections above. The same negative is shown for both antibodies as the primary antibodies shown were both raised in goat. n = 3; m. myometrium; s, stroma.

Mouse inter- and intra-implantation sites from day 5 of gestation (post-implantation) showed LIF and LIFR immunolocalization predominantly in decidual cells and the luminal epithelium (Figure 6A–F). LIF localized to the decidua (Figure 6B&C) and also to the the luminal epithelium (Figure 6C&D). LIFR localized to the decidua (Figure 6E–H).

### LIFR was upregulated in implantation sites in mice

**Figure 7:**
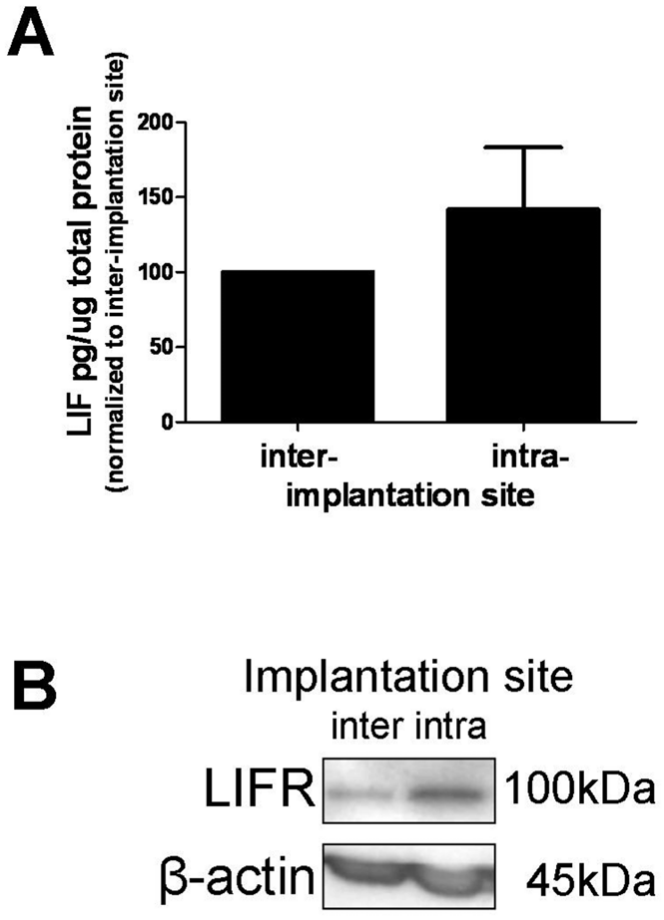
LIF and LIFR protein in murine inter- and intra-implantation sites. A. LIF protein in cellular extracts quantified by ELISA (inter- and intra-implantation sites from n = 3 independent animals). B. LIFR protein in cellular extracts quantified by Western blot (inter- and intra-implantation sites from n = 3 independent animals).

LIF protein was quantitated from inter-implantation (11.3±7.3 pg/µg) and intra-implantation (12.8±6.8 pg/µg) sites by ELISA. No significant difference was observed between inter-implantation and intra-implantation sites (Figure 7A) however the variance in LIF protein levels was quite high between mice (inter-implantation 3.0 to 18.1 pg/µg vs intra-implantation 6.7 to 20.1 pg/µg).

LIFR protein was up-regulated in intra-implantation sites compared to inter-implantation sites (Figure 7B).

### PEGLA impaired decidualization in mice

**Figure 8:**
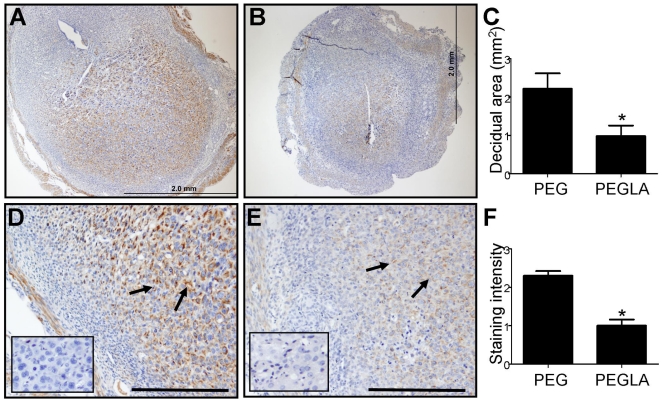
*In vivo* LIF inhibition impaired decidualization in mice. **A–B.** PEG (control) implantation sites (**A**; n = 5) had larger decidual areas (identified by desmin staining, brown) than PEGLA-treated implantation sites (**B**; n = 4). **C.** Graphical representation of the decidual area (identified by area of desmin staining) in implantation sites on D6. **D–E.** PEG control treated implantation sites (**D**; n = 5) showed stronger staining intensity of desmin (brown, arrows) than PEGLA-treated implantation sites (**E**; n = 4). **F.** Graphical representation of desmin staining intensity in decidual cells on D6. *. Significant difference between treatments, p<0.05; Scale 2.0 mm (A&B); 500 µm (D&E).

PEGLA administered by IP injection on D2 and D3 acts as a contraceptive by inhibiting attachment of the blastocyst to the uterine luminal epithelium [19]. Using PEGLA, we aimed to investigate the role of LIF during early decidualization *in vivo* in mice. A single dose of 900 µg (n = 2 PEGLA, n = 3 PEG) or 1200 µg (n = 2/group) PEGLA or PEG control at 10am D4 resulted in smaller decidual area (assessed by area of desmin staining: PEGLA 1.0±0.3 mm^2^, PEG 2.2±0.4 mm^2^, n = 4 PEGLA, 5 PEG; t_7_ 2.387, p<0.05; Figure 8A–C) and less intense desmin immunoreactivity of decidual cells (PEGLA 1.0±0.16, PEG 2.3±0.1, n = 5/group; t_8_ 6.500, p<0.05; Figure 8D–F) in PEGLA treated implantation sites compared to PEG control implantation sites. We have pooled the data from our two PEGLA treatments (900 µg and 1200 µg/injection) as no difference in decidual morphology was observed between the two doses.

## Discussion

Decidualization is a tightly regulated process, characterised by the sequential expression of regulatory genes [1]. A number of factors enhance progesterone-induced HESC decidualization and here we demonstrated that exogenous LIF enhanced MPA-induced decidualization of HESC *in vitro*. Further, LIF regulated IL6 and IL15 secretion by decidualizing HESC, identifying potential mechanisms of LIF action during HESC decidualization. Similarly in mice, we showed that administration of a LIF inhibitor post-implantation retarded decidualization. Both LIF and LIFR immunolocalized to decidual cells post-implantation in mice. This study showed that LIF is functionally important for decidualization in both human and murine endometrial stromal cells.

Previous studies have suggested that LIF protein production in the human uterus is mainly localized to the glandular epithelium [29], [30], [31], [32], however LIF-immunoreactive cells are localized to first trimester decidua [31], [32] as was found here. LIFR mainly localized to the epithelium, but was also present in decidualizing stromal cells [24] and 1^st^ trimester decidual cells.

Our in vitro data (mRNA expression of LIF in cultured HESC) suggests it is likely that the immunoreactive LIF in decidual cells is endogenously produced, however we were unable to detect secreted LIF protein by HESC in vitro (data not shown). We did not measure cellular LIF protein. Previous studies have shown that LIF is secreted from decidual biopsies, but not from isolated cells, either decidual or leukocyte [33] and estrogen induces the secretion of LIF by decidual cells isolated from first trimester decidua [32], however, these decidual cells are a lot further decidualized than the decidualizing HESC assayed here. Here, treatment of decidualizing HESC with LA alone did not affect decidualization, suggesting that any endogenous LIF produced (mRNA or protein) in our system was not active. Certainly, our decidualization stimulus (E+MPA) actually suppressed LIF mRNA expression in HESC compared to non-decidualized control. Interestingly, hCG, via prokineticin induces LIF mRNA in decidua [34], suggesting that it may be hCG which up-regulates decidual LIF production during pregnancy. Taken together, these data suggest that LIF is produced by decidual cells in vivo but that it is regulated by factors not present in our in vitro system.

Here for the first time we showed hormonal (E+MPA) regulation of LIFR mRNA in HESC. Hormonal regulation of LIFR occurs in sheep [35] and is likely also occuring in mice and hamsters where LIFR mRNA expression is up-regulated during decidualization whereas LIF is transiently expressed [5], [36], [37]. Interestingly, our data showed that the LIFR was up-regulated by the decidual stimulus: suggesting the decidualization stimulus ‘primed’ HESC to be responsive to LIF. Such ‘priming’ of decidualizing HESC to respond to local factors has also been demonstrated for total STAT3. STAT3 is present in decidualizing cells of the mid-late secretory phase [21] and 1^st^ trimester decidua [38] and our in vitro studies show that the decidual stimulus induces production of STAT3 protein by HESC [21], enhancing the ability of HESC to respond to cytokines such as IL11 and possibly LIF that signal via STAT3 activation. Taken together, this data suggests that the decidualization stimulus amplifies the response of HESC to LIF.

In a previous study, exogenous LIF had no effect on cAMP-induced HESC decidualization [8]. Medoxy-progesterone acetate (MPA; a stable synthetic progestin) was chosen for the present study as progesterone is the main physiological inducer of decidualization *in vivo* and other cytokines have been shown to progress progesterone-induced decidualization including IL11 and activin A whilst having no effect on cAMP-induced decidualization [12], [21]. cAMP and progesterone use different pathways during decidualization [10] and cAMP is thought to prime HESC to the action of progesterone [10], however both pathways are nevertheless required for the progression of normal decidualization [9].

We also examined the downstream actions by which LIF may regulate decidualization. First trimester decidual biopsies secreted high levels of G-CSF, GM-CSF, IL6, IL8 and MCP1 and correspondingly, decidualized HESC secreted high levels of IL6, IL8, IL15 and MCP1. It should be noted that decidual biopsies contain a large number of haematopoietic and other cells, thus this secretome represents more than just decidual secretions, however our data suggests that decidual cells may secrete IL6, IL8 and MCP1. Here, we report for the first time that LIF enhanced IL6 and IL15 secretion by decidualized HESC. IL15 is expressed by first trimester decidua [39], [40] and is up-regulated during P-induced HESC decidualization *in vitro* [40]. Implantation sites in IL15 null female mice lack decidual integrity as well as having an absence of uterine natural killer cells [41]. It remains to be determined whether the action of LIF on IL15 is direct or indirect and whether this also occurs in mice. LIF also up-regulated IL6 secretion by decidualizing HESC. IL6 has only been identified in the decidua in one previous study which used RNA arrays to investigate the effect of trophoblast conditioned media on stromal decidualization [42]. Future studies are required to determine nature of the interaction between LIF and IL6 and IL15.

In mice, decidualization progresses from the anti-mesometrial to the mesometrial region as stromal cells proliferate and enlarge [43]. We have shown LIF and LIFR protein localization to murine decidual cells and previous studies have shown the LIF signalling molecules STAT3 [44] and gp130 [37] are present in the mouse decidua. Intriguingly, LIFR mRNA (shown by in situ hybridization) was expressed by decidual cells just beneath the luminal epithelium on D5 of gestation [37] but our data suggests that the LIFR protein localized to decidualized cells throughout the implantation site. We confirmed the strong expression of LIFR in the decidua by Western blotting, where implantation sites, which by immunohistochemistry contained mainly decidual cells, had increased LIFR compared to inter-implantation sites, which contained no decidual cells.

Administration of our long-acting LIF antagonist (PEGLA) to mice during the initial stages of decidualization, which, unlike in women occurs only post-implantation in mice, resulted in reduced decidual area and reduced immuno-intensity of desmin, a decidual marker compared to control. Altogether, our data suggests that the LIF antagonist acted on decidual cells to suppress decidualization. Previous studies have indicated that LIF may enhance murine decidualization: LIF^−/−^ females cannot undergo artificially induced decidualization [14]. Intra luminal injection of a short-acting LIF inhibitor (LIF05, prior to implantation on D3) inhibited blastocyst attachment [15] and therefore decidualization, shown by a reduction in desmin filaments compared to control treated mice. This study also suggests that LIF acts via the luminal epithelium, whilst further demonstrating that LIF can also independently act via the decidual cells themselves to stimulate decidualization. Supporting this, intraluminal administration of LIF partially rescues the artifical decidual response in ovariectomized Foxa2 null mice which have an attenuated decidualization response [16]. Paradoxically, inhibition of LIF in cultured murine stromal cells undergoing *in vitro* decidualization enhances decidualization while exogenous LIF inhibits decidualization [17], suggesting that LIF may have an inhibitory effect on *in vitro* decidualization of murine stromal cells, opposite to *in vivo* studies. This study is the first to demonstrate that LIF is critical for decidualization following natural (LIF dependent) blastocyst attachment.

We have previously shown that PEGLA administration on D2 and D3 completely prevents implantation [19], thus, no decidual reaction is initiated. Using a short-acting LIF inhibitor Mohamet *et al* [15] showed that epithelial LIF signalling is critical for attachment only between mid-morning and mid-afternoon on D3. It is unlikely that PEGLA merely delayed implantation in our study as initial blastocyst attachment begins on D3.5 [28] and we did not begin injections until D4. Therefore PEGLA likely inhibited LIFR signalling post-implantation in the implantation site, leading to impaired decidual expansion.

All together, this study showed that LIF enhanced decidualization in both human and mouse models. We have identified a new role for LIF in implantation and suggest that LIF could be a useful target to either facilitate or block decidualization during the earliest stages of pregnancy.

## References

[1] Dimitriadis E, Menkhorst EM, Salamonsen LA, Paiva P (2010). Review: LIF and IL11 in trophoblast-endometrial interactions during the establishment of pregnancy.. Placenta.

[2] Dimitriadis E, White CA, Jones RL, Salamonsen LA (2005). Cytokines, chemokines and growth factors in endometrium related to implantation.. Human Reproduction Update.

[3] Paiva P, Menkhorst EM, Salamonsen LA, Dimitriadis E (2009). Leukemia inhibitory factor and interleukin-11: Critical regulators in the establishment of pregnancy.. Cytokine & Growth Factor Reviews.

[4] Bhatt H, Brunet LJ, Stewart CL (1991). Uterine expression of leukemia inhibitory factor coincides with the onset of blastocyst implanation.. Proceedings of the National Academy of Sciences USA.

[5] Yang Z-M, Le S-P, Chen D-B, Cota J, Siero V (1995). Leukemia Inhibitory Factor, LIF receptor, and gp130 in the mouse uterus during early pregnancy.. Molecular Reproduction and Development.

[6] Stewart CL, Kaspar P, Brunet LJ, Bhatt H, Gadi I (1992). Blastocyst implantation depends on maternal expression of leukaemia inhibitory factor.. Nature.

[7] Marwood M, Visser K, Salamonsen LA, Dimitriadis E (2009). Interleukin-11 and leukemia inhibitory factor regulate the adhesion of endometrial epithelial cells: implications in fertility regulation.. Endocrinology.

[8] Nakajima S, Tanaka T, Umesaki N, Ishiko O (2003). Leukemia inhibitory factor regulates cell survival of normal human endometrial stromal cells.. International Journal of Molecular Medicine.

[9] Gellersen B, Brosens J (2003). Cyclic AMP and progesterone receptor cross-talk in human endometrium: a decidualizing affair.. Journal of Endocrinology.

[10] Brosens JJ, Hayashi N, White JO (1999). Progesterone receptor regulates decidual prolactin expression in differentiating human endometrial stromal cells.. Endocrinology.

[11] Popovici RM, Kao L-C, Giudice LC (2000). Discovery of New Inducible Genes in in vitro Decidualized Human Endometrial Stromal Cells Using Microarray Technology.. Endocrinology.

[12] Jones RL, Salamonsen LA, Findlay JK (2002). Activin A promotes human endometrial stromal cell decidualization *in vitro*.. Journal of Clinical Endocrinology and Metabolism.

[13] Dimitriadis E, Robb L, Salamonsen LA (2002). Interleukin 11 advances progesterone-induced decidualization of human endometrial stromal cells.. Molecular Human Reproduction.

[14] Stewart CL (1994). The role of leukemia inhibitory factor (LIF) and other cytokines in regulating implantation in mammals.. Annals of the New York Academy of Sciences.

[15] Mohamet L, Heath JK, Kimber SJ (2009). Determining the LIF-sensitive period for implantation using a LIF-receptor antagonist.. Reproduction.

[16] Jeong J-W, Kwak I, Lee KY, Kim TH, Large MJ (2010). Foxa2 Is Essential for Mouse Endometrial Gland Development and Fertility.. Biology of Reproduction.

[17] Fouladi-Nashta AA, Andreu CV, Nijjar N, Heath JK, Kimber SJ (2004). Role of leukemia inhibitor factor (LIF) in decidualisation of murine uterine stromal cells in vitro.. Journal of Endocrinology.

[18] Menkhorst EM, Zhang J-G, Sims NA, Morgan PO, Soo P (2011). Vaginally administered PEGylated LIF antagonist blocked embryo implantation and eliminated non-target effects on bone in mice.. PLoS One.

[19] White CA, Zhang J-G, Salamonsen LA, Dimitriadis E (2007). Blocking LIF action in the uterus by using a PEGylated antagonist prevents implantation: a nonhormonal contraceptive strategy.. PNAS.

[20] Dimitriadis E, Stoikos C, Stafford-Bell M, Clark I, Paiva P (2006). Interleukin-11, IL-11 receptor [alpha] and leukemia inhibitory factor are dysregulated in endometrium of infertile women with endometriosis during the implantation window.. Journal of Reproductive Immunology.

[21] Dimitriadis E, Stoikos C, Tan Y-L, Salamonsen LA (2006). Interleukin 11 signaling components Signal Transducer and Activator of Transcription 3 (STAT3) and Suppressor of Cytokine Signaling 3 (SOCS3) regulate human endometrial stromal cell differentiation.. Endocrinology.

[22] Nilsson U, Johns TG, Wilmann T, Gao Y, Whitehead C (2010). Combination methotrexate and epidermal growth factor receptor inhibition as a novel medication-based cure of ectopic pregnancies.. Reproduction, Fertility and Development.

[23] Maslar IA, Riddick DH (1979). Prolactin production by human endometrium during the normal menstrual cycle.. American Journal of Obstetrics and Gynecology.

[24] Aghajanova L, Altmae S, Bjuresten K, Hovatta O, Landgren BM (2009). Disturbances in the LIF signalling pathway in the endometrium among women with unexplained infertility.. Reproductive Endocrinology.

[25] Fairlie WD, Uboldi AD, McCoubrie JE, Wang CC, Lee EF (2004). Affinity Maturation of Leukemia Inhibitory Factor and Conversion to Potent Antagonists of Signaling.. Journal of Biological Chemistry.

[26] Menkhorst E, Salamonsen LA, Robb L, Dimitriadis E (2009). IL11 antagonist inhibits uterine stromal differentiation, causing pregnancy failure in mice.. Biology of Reproduction.

[27] Simpson KJ, Ranganathan S, Fischer JA, Janssens PA, Shaw DC (2000). The gene for a novel member of the whey acidic protein family encodes three four-disulfide core domains and is asynchronously expressed during lactation.. The Journal of Biological Chemistry.

[28] Das SK, Lim H, Paria BC, Dey SK (1999). Cyclin D3 in the mouse uterus is associated with the decidualization process during early pregnancy.. Journal of Molecular Endocrinology.

[29] Aghajanova L, Stavreus-Evers A, Nikas Y, Hovatta O, Landgren BM (2003). Coexpression of pinopodes and leukemia inhibitory factor, as well as its receptor, in human endometrium.. Fertility and Sterility.

[30] Charnock-Jones DS, Sharkey AM, Fenwick P, Smith SK (1994). Leukaemia inhibitory factor mRNA concentration peaks in human endometrium at the time of implantation and the blastocyst contains mRNA for the receptor at this time.. Journal of Reproduction and Fertility.

[31] Cullinan EB, Abbondanzo SJ, Anderson PS, Pollard JW, Lessey BA (1996). Leukemia inhibitory factor (LIF) and LIF receptor expression in human endometrium suggests potential autocrine/paracrine function in regulating embryo implantation.. Proceedings of the National Academy of Sciences USA.

[32] Sawai K, Matsuzaki N, Okada T, Shimoya K, Koyama M (1997). Human decidual cell biosynthesis of leukemia inhibitory factor: regulation by decidual cytokines and steroid hormones.. Biology of Reproduction.

[33] Sharkey AM, King A, Clark DE, Burrows TD, Jokhi PP (1999). Localization of Leukemia Inhibitory Factor and Its Receptor in Human Placenta Throughout Pregnancy.. Biology of Reproduction.

[34] Evans J, Catalano RD, Brown P, Sherwin R, Critchley HOD (2009). Prokineticin 1 mediates fetal-maternal dialogue regulating endometrial leukemia inhibitory factor.. FASEB J.

[35] Song G, Satterfield MC, Kim J, Bazer FW, Spencer TE (2009). Progesterone and interferon tau regulate leukemia inhibitory factor receptor and IL6ST in the ovine uterus during early pregnancy.. Reproduction.

[36] Ding T, Song H, Wang X, Khatua A, Paria BC (2008). Leukemia inhibitory factor ligand-receptor signaling is important for uterine receptivity and implantation in golden hamsters (Mesocricetus auratus).. Reproduction.

[37] Ni H, Ding N-Z, Harper MJK, Yang Z-M (2002). Expression of leukemia inhibitory factor receptor and gp130 in mouse uterus during early pregnancy.. Molecular Reproduction and Development.

[38] Garcia MG, Tirado-Gonzalez I, Handjiski B, Tometten M, Orsal AS (2007). High expression of survivin and down-regulation of Stat-3 characterize the feto-maternal interface in failing murine pregnancies during the implantation period.. Placenta.

[39] Kitaya K, Yasuda J, Yagi I, Tada Y, Fushiki S (2000). IL-15 Expression at Human Endometrium and Decidua.. Biology of Reproduction.

[40] Okada S, Okada H, Sanezumi M, Nakajima T, Yasuda K (2000). Expression of interleukin-15 in human endometrium and decidua.. Molecular Human Reproduction.

[41] Ashkar AA, Black GP, Wei Q, He H, Liang L (2003). Assessment of Requirements for IL-15 and IFN Regulatory Factors in Uterine NK Cell Differentiation and Function During Pregnancy.. The Journal of Immunology.

[42] Hess AP, Hamilton AE, Talbi S, Dosiou C, Nyegaard M (2007). Decidual stromal cell response to paracrine signals from the trophoblast: Amplification of immune and angiogenic modulators.. Biol Reprod.

[43] Abrahamsohn PA, Zorn TMT (1993). Implantation and decidualization in rodents.. The Journal of Experimental Zoology.

[44] Teng CB, Diao HL, Ma XH, Xu LB, Yang ZM (2004). Differential expression and activation of Stat3 during mouse embryo implantation and decidualization.. Molecular Reproduction and Development.

